# A practical guide to optical coherence tomography angiography interpretation

**DOI:** 10.1186/s40942-020-00262-9

**Published:** 2020-11-13

**Authors:** Eugenia Custo Greig, Jay S. Duker, Nadia K. Waheed

**Affiliations:** 1 New England Eye Center, Tufts Medical Center, 800 Washington Street, Box 450, Boston, MA 02111 USA; 2 Yale School of Medicine, New Haven, CT USA

**Keywords:** Optical coherence tomography angiography, Interpretation, Artifacts

## Abstract

**Background:**

Optical coherence tomography angiography (OCTA) can image the retinal vasculature in vivo, without the need for contrast dye. This technology has been commercially available since 2014, however, much of its use has been limited to the research setting. Over time, more clinical practices have adopted OCTA imaging. While countless publications detail OCTA’s use for the study of retinal microvasculature, few studies outline OCTA’s clinical utility.

**Body:**

This review provides an overview of OCTA imaging and details tips for successful interpretation. The review begins with a summary of OCTA technology and artifacts that arise from image acquisition. New methods and best practices to prevent image artifacts are discussed. OCTA has the unique ability among retinovascular imaging modalities to individually visualize each retinal plexus. Slabs offered in standard OCTA devices are reviewed, and clinical uses for each slab are outlined. Lastly, the use of OCTA for the clinical interpretation of retinal pathology, such as diabetic retinopathy and age-related macular degeneration, is discussed.

**Conclusion:**

OCTA is evolving from a scientific tool to a clinical imaging device. This review provides a toolkit for successful image interpretation in a clinical setting.

## Background

Optical coherence tomography angiography (OCTA) first became commercially available in 2014 [1]. This technology allowed for the visualization of retinal microvasculature in vivo. Prior to OCTA, fluorescein angiography (FA) and indocyanine green angiography (ICGA) were the mainstay modalities for retinovascular visualization. These imaging modalities generated a 2-dimensional en face view of the retinal vasculature and did not allow for the individual visualization of retinal capillary plexuses. OCTA offered in vivo visualization of the retinal microvasculature in a depth-resolved fashion, without the need for time-consuming dye administration.

Prior to its commercial release, OCTA was available as a research tool. Investigational groups explored the utility of OCTA in a wide range of ocular pathologies including age related macular degeneration (AMD), diabetic retinopathy (DR), and uveitis [2–8]. A large body of literature emerged to describe vascular findings on OCTA. Though scientifically novel and relevant to the understanding of ocular pathophysiology, these studies often reference language and metrics that remain inaccessible to clinical ophthalmologists. This review will offer a basic toolkit for OCTA interpretation in clinical practice and explore its utility in assessing retinal pathology.

### Functioning principles

In 1991 optical coherence tomography (OCT) was described in the literature [9]. This novel imaging modality allowed for detailed visualization of the posterior retina in a cross sectional fashion. This non-invasive imaging technique changed the clinical management of many retinal diseases.

Though original clinical OCT units were based on time domain detection, spectral domain OCT (SD-OCT) is now the clinical standard. This technology utilizes a light beam that is split into an exploring arm and a reference arm to generate an image. Light from the exploring arm is reflected, absorbed or scattered as it passes through the structures of the posterior retina. The light from the exploratory arm is then compared to the undisturbed light path of the reference arm. The difference between the two light paths yields information regarding the depth and brightness of structures within the retina. Each data point generated is referred to as an A-scan. As the device travels transversely through tissue it collects a series of A-scans that are compiled together into a retinal cross-section known as a B-scan. A collection of B-scans produces a three-dimensional representation of the retina that can be used to interpret depth-resolved structural anatomy [10].

OCTA was developed as an extension of OCT imaging. OCTA technology utilizes motion contrast to detect blood flow. When two successive images of a scene are taken, stationary objects will not change, while moving objects will become apparent. OCTA captures successive A-scans at the same retinal location, and each scan capture is separated by a brief lapse in time. As light is reflected back, a difference in signal will be detected between the two scans. This difference is due to motion between the scans and is termed decorrelation signal. Because the retina is a static tissue, the decorrelation signal is attributed to the movement of blood through the retinal vasculature. Thus, a decorrelation map is generated that mirrors the flow of blood in the back of the eye, rendering a representation of its vascular networks.

The time delay between each B-scan captured is termed interscan time. Different interscan times allow for the detection of specific blood flow speeds. For OCTA technology to detect blood flow within a vessel, erythrocytes must move within the length of the OCT beam between successive scans. A short interscan time captures the movement of a fast-moving erythrocyte before it exits the length of the OCT beam. Short interscan times, therefore, improve the visualization of fast-moving vascular beds. Increasing the interscan time allows slow-moving erythrocytes a greater chance to move between successive scans, increasing the sensitivity to slow-moving vascular beds [11]. However, this increased sensitivity comes at the cost of increased image artifacts secondary to involuntary eye motion. Though interscan time manipulation has led to interesting discoveries regarding retinal vascular flow, commercial OCTA devices do not allow for interscan time adjustment [5]. Instead, OCTA manufacturers utilize preset interscan times to deliver an image that displays flow and minimizes artifact. In summary, OCTA is not a representation of retinal vessels, but of retinal blood flow within a predetermined range [12].

### Spectral domain and swept source OCTA

The two most commonly available OCTA devices are spectral domain and swept source devices. These devices vary in their light source, as well as their light detectors. Spectral domain OCTA (SD-OCTA) utilizes a near-infrared super luminescent diode with a wavelength of ~ 840 nm as a light source, and a spectrometer as a detector. Swept Source OCTA (SS-OCTA) utilizes a tunable swept laser with a wavelength of ~ 1050 nm as a light source and a single photodiode as a detector [1]. The increased wavelength of swept source devices is safer for the eye and permits the use of a high power laser. The faster scanning speed of swept source devices allows for larger scan areas that visualize a greater portion of the posterior retina, as well as dense scanning patterns that increase detail visualization [13]. Overall the most clinically significant difference between the two devices is the longer wavelength of the swept source device. The increased wavelength of the swept source device reduces its axial resolution when compared to SD-OCTA [14]. However, longer wavelengths allow for improved penetration through the retinal pigment epithelium (RPE) and better visualize the choroid [15]. Regardless of this added benefit, swept source devices are costly, making SD-OCTA technology more widespread [10].

### Strengths of OCTA

OCTA displays in vivo retinal vasculature in a depth-resolved fashion. Segmentation of volumetric data leads to identification of retinal capillary plexuses individually, providing detail and resolution similar to that of histologic studies [16]. Further, OCTA provides improved visualization of the deep capillary plexus and choroid when compared to FA and ICGA [17]. OCTA does not utilize dye to visualize vessels which confers it with multiple advantages. First, OCTA generates high-contrast images that are not obscured by dye leakage from vessels, leading to more pronounced definition of retinal vasculature. Second, this dye–free imaging modality does not expose patients to the risks associated with contrast dye, which range from mild allergic reactions to anaphylaxis [18]. Patients that have relative contraindications to dye imaging, including those with renal failure or poor intravenous access, can undergo OCTA imaging without limitations.

OCTA imaging is fast and thus useful for patients that require recurrent vascular imaging, such as those being treated with vascular endothelial growth factor antagonists (anti-VEGF) for wet AMD or diabetic macular edema (DME). Though FA remains the gold standard for imaging of the retinal vasculature, OCTA was found to be of great utility for detecting complications of retinal vein occlusion, DR and AMD [19–21].

### Limitations of OCTA

Beyond its many advantages OCTA has a number of limitations. As aforementioned, flow detection on OCTA requires scanning a single location multiple times. This increases imaging times, especially for larger scan areas. Newer devices with faster scanning speeds have shorter image acquisition times, but capturing large amounts of data at fast speeds translates into slower processing and saving times.

Furthermore, device hardware and software are highly variable between OCTA manufacturers. Each device is programmed with a proprietary algorithm to detect flow. The difference between these algorithms is highly technical and beyond the scope of this discussion. However, clinicians must be aware that such differences exist, as they can affect imaging results. When possible, patients should be imaged on the same device to assure accurate comparison between visits. Moreover, image size can affect the degree of vascular detail captured [22]. The A-scan density for each scan size differs, affecting the visualization of fine vessels. Vessels that are clearly visualized on a 3 × 3 mm scan might not be as salient on a 6 × 6 mm scan, creating the illusion of vessel drop-out when the true difference lies in image resolution. Consistent imaging with the same device and scan size can improve vascular tracking overtime.

Lastly, though dye-free OCTA imaging is safer for patients, it has some disadvantages. OCTA cannot visualize dye leakage, a common landmark of inflammatory vascular pathology and a sign of blood-retinal barrier breakdown. OCTA cannot provide information on transit time or vascular filling either. Moreover, ultra-wide field fundus photography allows FA to capture vascular detail in the retinal periphery. Though newer OCTA devices have larger scan patterns available, the ability of OCTA to detect pathology in the retinal periphery, such as peripheral non-perfusion, remains limited.

## OCTA interpretation pitfalls

OCTA is a powerful technology that provides a wealth of information, yet adequate image interpretation requires an understanding of the platform’s inherent shortcomings. OCTA imaging is vulnerable to artifacts that arise from image acquisition and processing. Artifacts can lower image quality and create the illusion that a spurious feature generated during image acquisition is part of the tissue being imaged. The next section will describe common OCTA artifacts and the tools available to correct them.

### Signal strength

OCTA image quality is largely dependent on a high “signal-to-noise” ratio. Signal is defined as the information derived from the tissue being imaged. Noise is the spurious, unwanted information generated through image acquisition and processing. Image quality drops if signal levels are low or if noise levels are high. A common example of signal reduction is media opacity secondary to cataracts. Cataracts reduce light penetration through the vitreous as well as the signal produced by retinal tissue, the result is a reduction in signal-to-noise ratio and overall image quality. Unlike cataracts, which cannot be modified prior to image acquisition, other factors that reduce image signal, such as dry-eyes and patient positioning, can be easily overcome with proper lubrication and trained imaging professionals.

On most platforms built-in device software reports a signal strength score for each acquisition. Though algorithms used to determine this score are proprietary and differ between devices, signal strength scores provide a quick way to decide whether to attempt interpretation or reacquire the image.

### Motion artifact

**Fig. 1:**
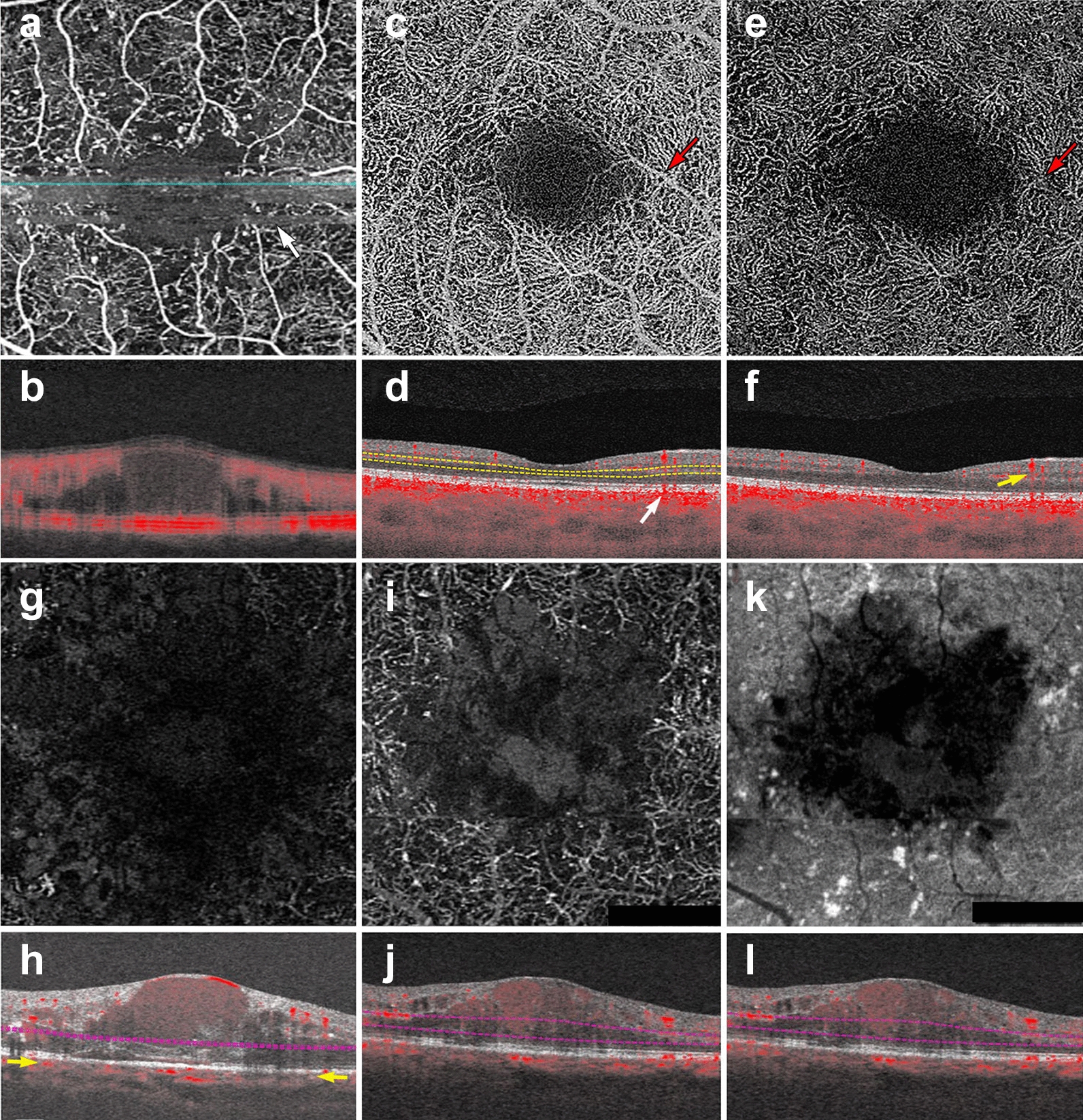
OCTA Artifacts. **a**, **b** *Motion artifact*: en face image of the full retinal projection, white arrow points to an example of motion artifact (**a**). The horizontal lines represent areas where OCTA acquisition was disrupted by movement. B-scan through this area (**b**) shows a blurry image with erroneous flow pixels. **c**–**f** *Projection artifact*: en face projection of the ddeep retinal layer before artifact removal **c** and after artifact removal **e**. Red arrows point to a projected vessel from the superficial retinal layer, note that the same vessel is no longer present after software correction. Corresponding B-scans (**d**, **f**) show a superficial retinal vessel reflected on the RPE (white arrow) and a decorrelation tail trailing below the vessel (yellow arrow). **g**, **h** *Segmentation error*: en face projection of the choriocapillaris in a patient with diabetic macular edema. At first glance, lack of flow pixels suggests widespread non-perfusion (**g**), however, B-scan reveals a significant segmentation error due to edema (**h**). Yellow arrows point to correct choriocapillaris segmentation level. **i**–**l** *Shadowing:* en face projection of the deep capillary plexus suggests central vascular loss (**I**). Corresponding structural en face image shows reduced signal in this area (**k**). Accompanying B-scan reveals signal attenuation is secondary to overlying edema, not vascular loss (**j**, **l**)

As explained earlier, OCTA deduces information from the motion of blood cells relative to the surrounding static retinal tissue. During imaging sessions patients may move their head, neck and eyes. This type of motion is referred to as bulk motion. Since OCTA attempts to derive information from movement, any movement that is not related to red blood cells can be misinterpreted as flow. Thus, bulk motion creates a type of artifact termed “motion artifact”. On an en face image, motion artifacts appear as horizontal lines through the scan (Fig. 1a). This is because movement has impeded proper data collection in that area. A cut through the artifact will show continuous, strong flow. This false flow is secondary to the detection of eye movement, not vascular flow (Fig. 1a, b).

Many device manufacturers rely on two types of technology to correct for motion artifact: mechanical eye tracking and software motion correction [1]. Eye tracking measures the position of the eye and corrects for eye movement when the change in eye position exceeds a predetermined threshold. Devices can rescan the area of detected motion and incorporate it into the original image [23]. Software motion correction obtains a horizontal and a vertical raster scan [24]. The software interprets the two scans together to calculate displacement between A-scans and corrects for motion in the final image. Today, most devices combine both technologies to produce high quality images.

### Shadowing artifact

Shadowing occurs when the OCT beam is blocked and cannot reach the outer retinal layers. Many pathological features cause shadowing including drusen, hemorrhage and vitreous floaters [17]. Light is reflected off of these structural aberrations and does not penetrate the underlying tissue. Areas below these features appear as flow voids because the OCT beam cannot adequately assess them (Fig. 1g–j). The longer wavelength of SS-OCTA systems makes them less prone to shadowing than their spectral domain counterparts. SS-OCTA devices can assess the outer retinal layers below these features, though some signal attenuation persists. Recently, several groups have published algorithms that combine structural and angiography data to compensate for shadowing artifact below drusen [25, 26]. Though this technology is not currently available on commercial OCTA devices, this research shows promise and similar algorithms could be incorporated into future software updates.

### Projection artifacts

Projection artifacts, or decorrelation tails, are perhaps the most prominent and important artifacts seen on OCTA. As the OCT beam penetrates the retina, it first encounters the superficial capillary plexus. A portion of light is reflected back from this plexus and interpreted by the device. Light that is not reflected continues to travel through the retina until it reaches the RPE. The RPE acts a natural reflector, reflecting light back towards the OCTA device. Light that arrives at the RPE is influenced by the flow above it and generates a decorrelation signal that mimics superficial vascular beds. The result is the erroneous appearance of superficial vessels in the outer retina and RPE (Fig. 1c, d). This misinterpretation of flow is termed projection artifact.

Device manufacturers have engineered many ways to overcome projection artifacts (Fig. 1e). The first form of projection artifact removal (PAR), slab-subtraction, subtracted inner retinal vascular patterns from outer retinal slabs [27]. Though this algorithm succeeded in reducing projection artifact, it also led to signal loss in the outer retinal layers. Further, slab-subtraction depended on pre-determined slab selection and did not successfully suppress projection artifacts on B-scan. In 2016, Zhang and colleagues published a projection-resolved PAR algorithm. Unlike slab-subtraction, this new algorithm worked on a voxel-by-voxel basis, utilizing intensity-normalized decorrelation values to distinguish in situ flow from projection artifact. This algorithm successfully identified the presence of multiple in situ vessels along an axial scan and did not suppress flow signal in the outer retina. Such improvements allowed for the visualization of three distinct retinal vascular plexi on en face and B-scan [28]. More recently, Garrity et al. proposed a three-dimensional PAR algorithm. Similar to the projection-resolved technique described by Zhang’s group, this new algorithm determines in situ flow by assessing intensity-normalized decorrelation signals on a voxel-by-voxel basis. However, the three-dimensional model incorporates signal intensity information along the Z-axis, assessing OCTA signal in all directions around a single voxel prior to determining if true flow is present [29, 30].

Though technological innovation has greatly improved PAR, compensation methods are not perfect, nor are they uniformly available across OCTA devices. When interpreting OCTA images, the easiest way to detect projection artifacts is to compare en face flow with the corresponding B-scan. On B-scan, projections artifacts appear as “tails” arising from overlying vessels and should not be interpreted as true flow (Fig. 1f).

### Segmentation artifacts

The review software on commercially available devices provides a series of retinal slabs for analysis (i.e. full retinal, superficial retina, choriocapillaris, choroid). Each slab is generated by selecting an upper and lower boundary that slices horizontally through the three-dimensional OCTA cube. The vessels that lie within these two boundaries are projected to produce an en face image. Algorithms automatically detect predetermined retinal boundaries based on distinguishing characteristics such as reflectivity and texture [1]. However, segmentation algorithms were developed by imaging healthy eyes with distinct retinal layers [31]. Disease pathology, such as fluid seen in DME, drusen in AMD and even myopia, can drastically alter retinal anatomy and cause algorithms to mislabel boundaries (Fig. 1g, h). In such cases, assessing segmentation on the B-scan can reveal the error.

There are two ways to manipulate segmentation boundaries in order to correct for segmentation errors: slab selection and manual segmentation. Slab selection is simple and involves dragging a preset boundary up and down the z-axis. This action generates larger or smaller slabs to better assess pathology of interest (i.e. type 2 macular neovascularization in wet AMD). Manual segmentation allows the user to retrace the mislabeled retinal layer (i.e. Bruch’s membrane in a patient with drusen). Proper retracing must be completed for each B-scan which is a time consuming and laborious process. This type of boundary correction is largely reserved for research purposes, as it lacks utility in the time constraints of clinical practice.

## OCTA interpretation: basic toolkit

To ensure correct interpretation, the three-dimensional quality of OCTA imaging must be respected. Viewing two-dimensional OCTA representations without simultaneously interacting with the three-dimensional dataset can confound interpretation. As a general rule, three images must be concurrently assessed for accurate OCTA interpretation: the en face OCTA image; the corresponding B-scan with flow overlay and segmentation lines displayed; and the structural en face intensity image for the selected slab. Assessing all three image components at once reduces erroneous findings.

**Fig. 2:**
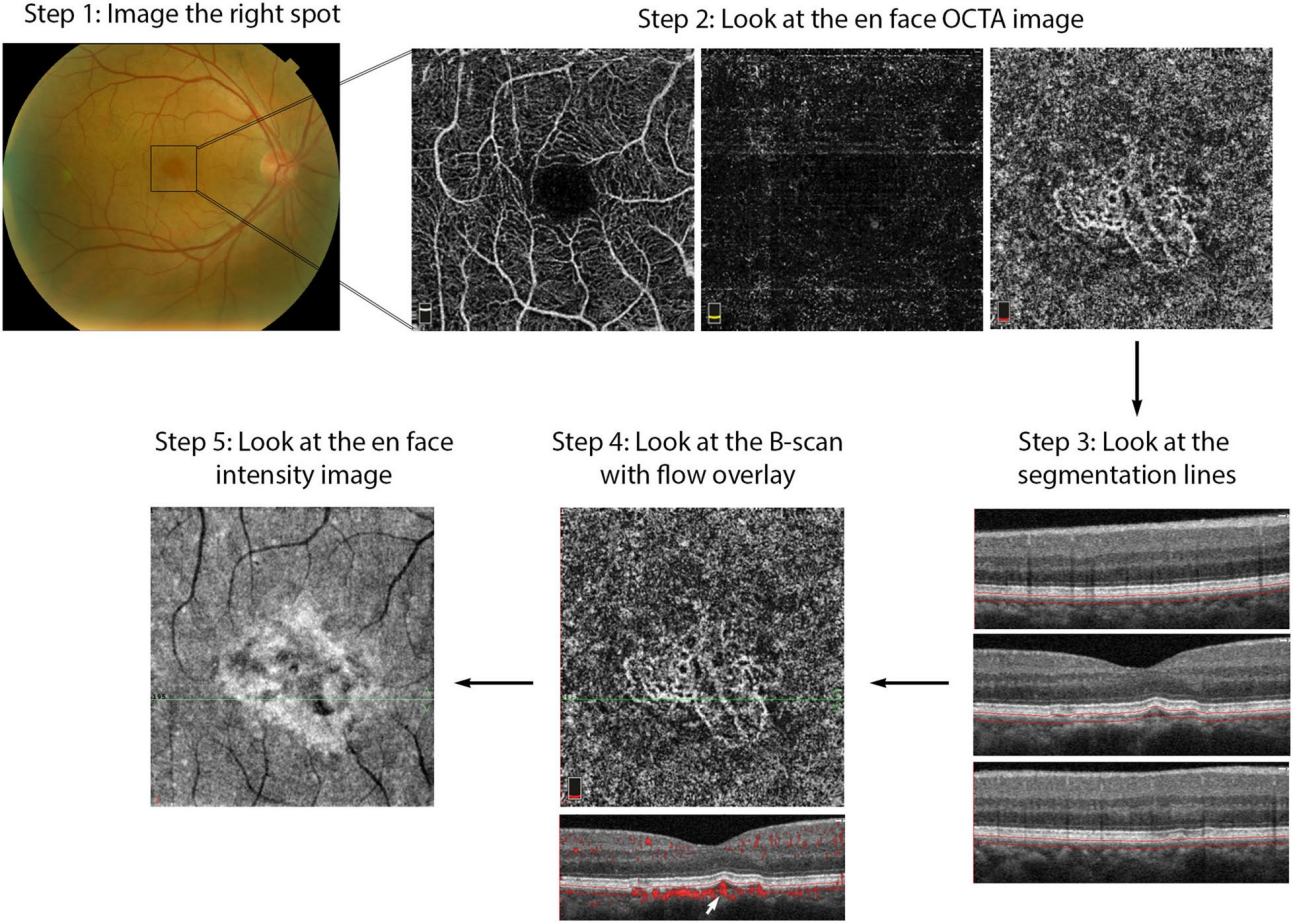
OCTA Interpretation Toolkit. How to apply step-by-step OCTA interpretation toolkit in a patient with AMD. *Step 1: Image the right spot*. Color fundus photograph of a patient with known AMD, the macula was selected as the area of interest and imaged. *Step 2: Look at the en face OCTA images*. 3 × 3 mm en face projections of each the full retinal depth, the avascular slab and the choriocapillaris (from left to right). The avascular and choriocapillaris slabs should be assessed in this AMD patient to check for presence of MNV. Note clearly defined vascular structures in the choriocapillaris slab (right most image), this is concerning for a type 1 MNV. *Step 3: Look at the segmentation lines.* Segmentation is assessed at multiple points throughout the slab to ensure accuracy. *Step 4: Look at the B*-*scan with flow overlay.* B-scan through lesion shows clear flow below the RPE (white arrow) and no projection artifact from overlying vasculature, suggesting this is in fact an MNV. *Step 5: Look at the en face intensity image.* En face intensity image for the choriocapillaris slab shows strong signal, shadowing artifact is not expected to disrupt image interpretation. *Summary:* Methodical OCTA image analysis uncovered a type 1 MNV that was not visible on fundus examination or on full depth retinal projection

The following section describes a step-by-step approach to OCTA interpretation (Fig. 2). The steps described can be applied to any device and to any type of ocular pathology. The model provides a foundation for OCTA interpretation and helps users avoid common mistakes.

### Step 1: image the correct spot

The first step in proper OCTA interpretation is to ensure the right location in the posterior retina is imaged. OCTA devices offer a range of scan sizes that can be centered at the macula and, in a subset of devices, the optic nerve head. To increase the scanning area, manufacturers developed montage options that stitch multiple scans together into a single image. Though montage images display a large portion of the posterior retina, the repeated imaging they require is time consuming and can be tiring for patients. Given decreased resolution in larger scan sizes, it is preferable to select the smallest scan size possible to visualize the pathology of interest. For instance, a small MNV can be visualized with greatest detail in a 3 × 3 mm scan. However, in a patient with DR, a larger scan size might be more appropriate for the assessment of proliferative diabetic retinopathy (PDR) and neovascularization elsewhere (NVE).

When the macular scan does not capture desired pathology, internal fixation targets can be moved to visualize different sectors of the posterior pole. For example, in the case of choroidal hemangiomas, lesions often occur far from the macula and might be missed by a macular scan [32]. Moving internal fixation targets, and employing external fixation targets when necessary, can help imaging technicians capture more peripheral lesions. For OCTA imaging to be effective, the clinician should first highlight what area of the posterior retina contains the pathology of interest. This ensures the technician will image the right location, allowing full visualization of desired pathology.

### Step 2: look at the en face OCTA image

En face OCTA images provide a valuable overview of the retinal vasculature. These images can display a vascular projection of the full retinal depth or separate projections of each the superficial capillary plexus, the deep capillary plexus, the avascular retina, the choriocapillaris and the choroid.

**Fig. 3:**
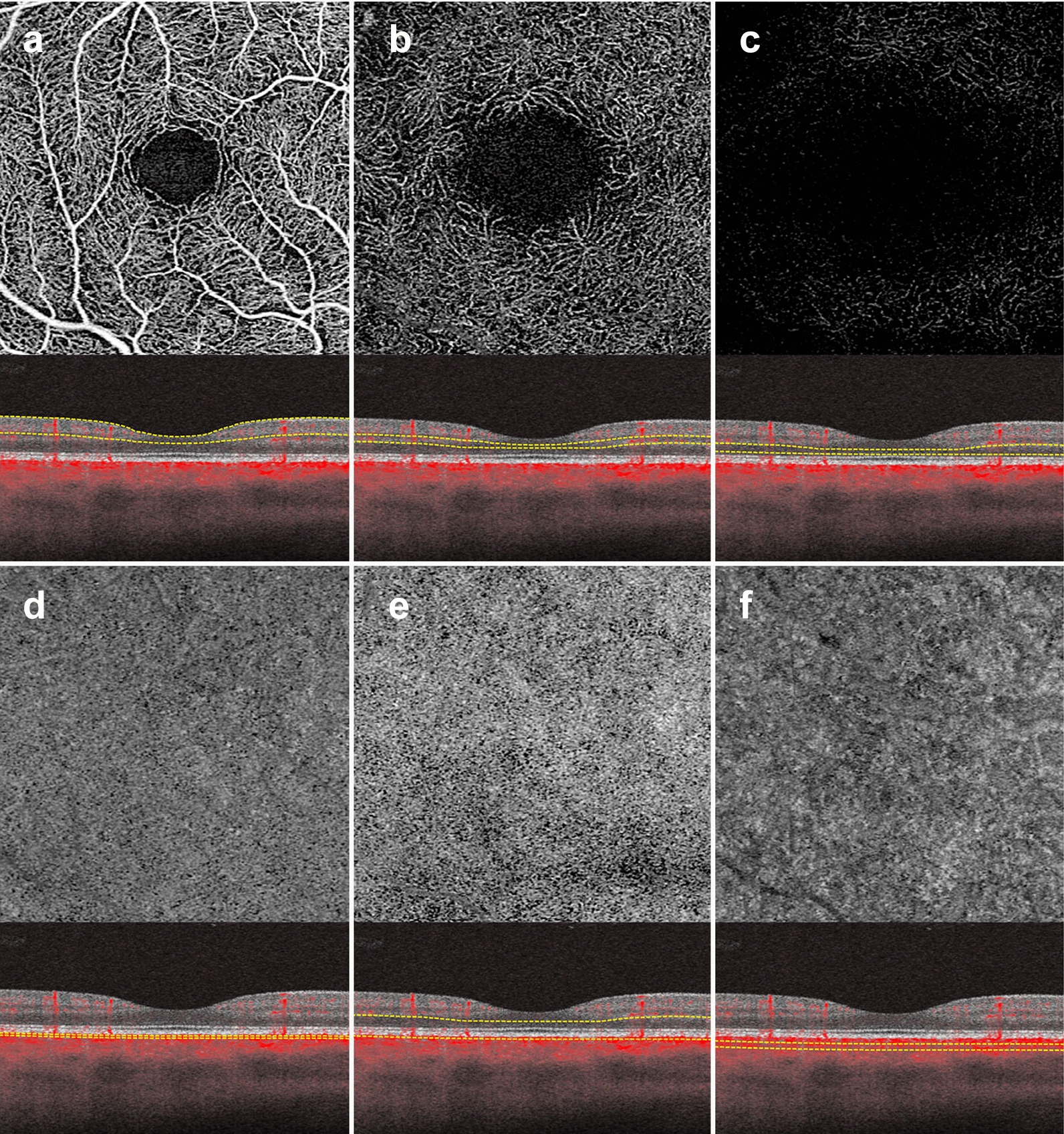
OCTA slabs and corresponding segmentation boundaries. **a** *Superficial retinal slab*. This slab displays information for the internal limiting membrane, the ganglion cell layer and the inner plexiform layer. It is useful for FAZ area delineation in diabetic retinopathy. **b** *Deep retinal slab.* This slab displays information for the inner nuclear layer and the outer plexiform layer. Projection artifact removal was used to block signal from the superficial capillary plexus on this en face image. This slab may show the first signs of vascular loss in diabetic retinopathy. **c** *Avascular slab*. This slab displays information for the outer plexiform layer and the outer nuclear layer. Note that despite projection artifact removal remnants of overlying vessels are noted in this image. **d** *Choriocapillaris slab.* Along with the avascular slab, the choriocapillaris slab is important for detection of macular neovascularization in AMD. **e** *Outer retinal choriocapillaris slab.* This slab displays information for the outer plexiform layer, the outer nuclear layer and the choriocapillaris. It is useful as a screening tool for all types of macular neovascularization in AMD. **f** *Choroid slab.* Clear choroidal vessels are not visualized due to signal blockade from the RPE and overlying retinal layers

The automated superficial retinal slab in most devices spans the internal limiting membrane, the ganglion cell layer and the inner plexiform layer (Fig. 3a). This slab captures the superficial capillary plexus of the retina and, often, part of the radial peripapillary capillary plexus. It also delineates the foveal avascular zone. This slab is important in retinal vascular diseases where drop out or malformation of the microvasculature can be seen. In DR, for example, enlargement of the foveal avascular zone, capillary drop out, and microaneurysms are best visualized on this slab. Additionally, some devices allow for quantitative evaluation of the vessels in this slab including vessel density and foveal avascular zone area.

On most devices, the deep retinal slab spans the inner nuclear layer and the outer plexiform layer and includes the intermediate and deep capillary plexuses (Fig. 3b). OCTA is the only clinically available commercial imaging modality that can visualize these two capillary plexuses in vivo [1]. Studies have shown that the deep capillary plexus is not visible on FA due to its intraretinal depth and the small size of its vessels [29]. The deep capillary plexus may be one of the first vascular beds to be affected in patients with retinal vascular diseases such as diabetes [33, 34]. In fact, OCTA studies of the deep capillary plexus have shown early changes associated with DR pathophysiology [34]. However, analysis of the deep retinal plexus must be conducted with care, as the presence of projection artifacts from the superficial plexus and the software used to remove these artifacts can often confound interpretation.

The avascular or outer retina slab spans the outer plexiform layer and the outer nuclear layer. This slab is avascular in healthy individuals (Fig. 3c). The presence of vessels in this layer is usually an indicator of pathology. For example, in patients with wet AMD the avascular layer may show type 2 or type 3 macular neovascularization (MNV). Though this layer should always be evaluated in patients with suspected AMD, it is prone to segmentation errors caused by outer retinal pathology such as a pigment epithelial detachments (PEDs).

The choriocapillaris slab is defined as a 20-micron slab below the RPE (Fig. 3d). Much like the avascular slab, the choriocapillaris slab should be evaluated in AMD and other pathologies associated with MNV formation. The choriocapillaris slab will not show distinct vascular channels. Instead, flow is represented on a pixel-by-pixel basis with hyper-reflective spots representing flow in this vascular layer. Presence of well-formed vascular channels in this slab (especially between Bruch’s membrane and the RPE) are very suspicious for choroidal neovascularization, specifically for type 1 MNV.

Due to the utility of the avascular and choriocapillaris slabs for MNV detection, some instruments display a combination of both slabs, known as the outer retinal and choriocapillaris (ORCC) slab (Fig. 3e). First described by Rosenfeld et al., this slab spans the outer plexiform layer, the outer nuclear layer and the choriocapillaris and can be used as a screening slab for type 1, 2 or 3 MNVs [13].

Lastly, the choroid slab displays a 50-micron slice posterior to the choriocapillaris (Fig. 3f). Differences in wavelength and light source lead to variable detection of choroidal vasculature between devices. Advances in laser technology allow for deeper penetration and visualization of choroidal vessels in investigative prototype models [35]. Though this technology is not yet commercially available, future device iterations might improve choroidal visualization and provide insight into pathologic changes occurring in this vascular layer.

### Step 3: look at the segmentation lines

Pre-set slabs in OCTA devices are generated by automated segmentation. Retinal layer detection is remarkably accurate in healthy eyes, but can be distorted in the presence of retinal pathology [31]. Once a slab is selected for interpretation, it is important to assess the segmentation lines that define it to ensure correct identification of retinal boundaries. Improper slab placement can lead to pathology being missed or to the mislabeling of normal vasculature as pathologic. For instance, in AMD, inaccurate slab selection might cause an MNV to be missed or, contrastingly, it might cause a PED to be confused for an MNV. If pathology has in fact distorted the software’s ability to detect retinal layers, retinal slab selection can be coarsely adjusted by dragging the slab boundaries up or down.

### Step 4: look at the B-scan with flow overlay

OCTA displays a two-dimensional projection of a three-dimensional structure, the retina. En face images should, therefore, always be interpreted in the context of their corresponding B-scan. For instance, en face projection can erroneously depict two vessels traveling at different retinal depths as a single branching vessel. Simultaneous assessment of the B-scan cross section provides a depth reference for the vasculature seen on en face and can help discern these optical illusions. Moreover, B-scan analysis can uncover projection artifacts. When assessing flow in the outer retinal layers, B-scan images can distinguish true flow from decorrelation tails produced by overlying vasculature. B-scans are critical when assessing pathology because they can help correlate vasculature to pathologic structural changes. For example, identification of flow between the RPE and the Bruch’s membrane in the presence of a PED gives a high level of confidence for the presence of a type 1 MNV (Figs. 2, 9).

### Step 5: look at the en face intensity image

If light penetration through the retina is obfuscated by overlying pathology, such as fluid, hemorrhage or drusen, flow cannot be adequately assessed. The presence of these pathologic features reduces the signal produced by underlying retinal layers. As explained earlier, this reduction in signal is termed shadowing and can be misinterpreted as reduced flow.

**Fig. 4:**
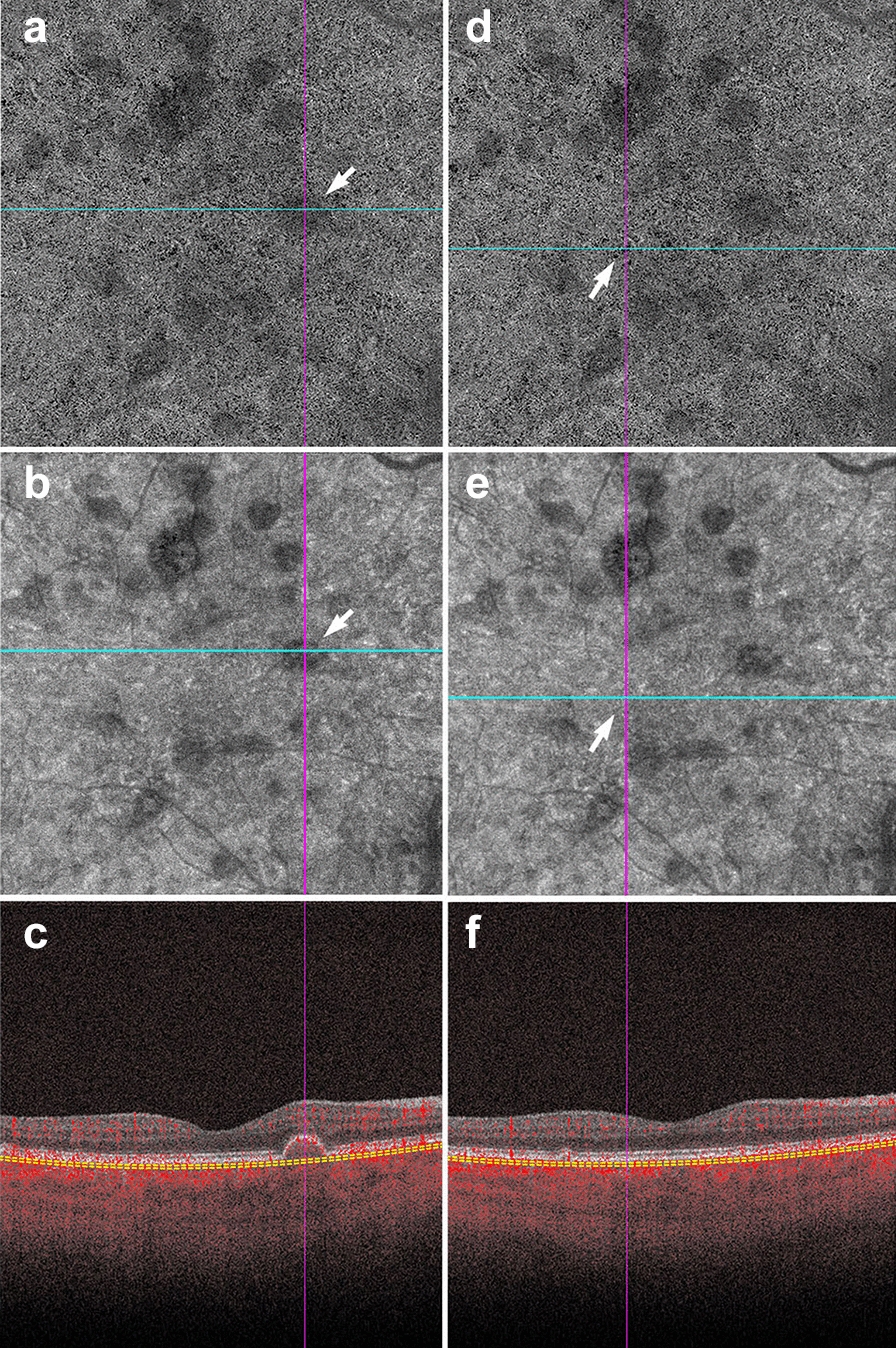
Shadowing in AMD. **a–f** Signal attenuation from drusen can be misinterpreted as non-perfusion in the choriocapillaris. Careful examination of the corresponding structural OCTA scan can rectify this error. **a**, **d** 6 x 6 mm choriocapillaris slab of a patient with AMD. **a** White arrow points to an area of apparent flow reduction. **b** Corresponding structural choriocapillaris slab. White arrow shows that the same area has reduced signal intensity. **c** B-scan shows a druse at this location. Signal reduction in this area should not be interpreted as non-perfusion, as it is likely due to signal attenuation and not flow reduction. **d** Identical choriocapillaris slab as that shown in panel A. White arrow points to an area of apparent flow reduction. **e** Corresponding structural choriocapillaris slab shows that this area has appropriate signal intensity (white arrow). **f** B-scan shows no druse or underlying pathology in this area. Signal reduction in this area can be appropriately interpreted as choriocapillaris loss

Assessing the en face intensity image in concert with the OCT angiogram can help determine if loss of flow is real, or mere shadowing from overlying pathology. The structural and angiography projection for a given retinal slab should be examined in concert. If the structural en face image shows good signal intensity and the angiogram shows reduced flow in the same area, this can be interpreted as a true reduction in flow. If the structural en face image shows low signal intensity and the angiogram shows reduced flow in the same area, shadowing artifact is likely (Fig. 4).

**Fig. 5:**
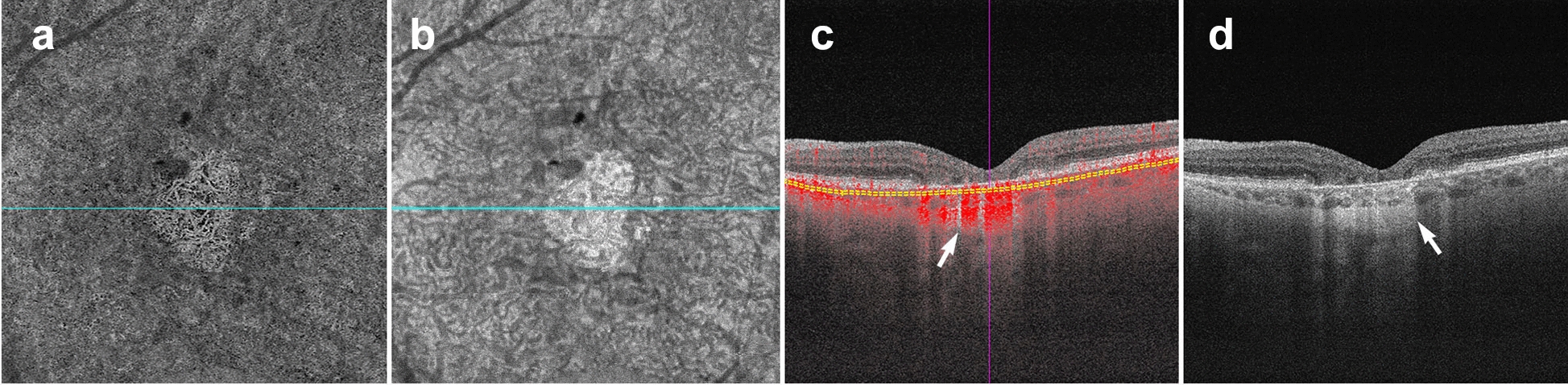
Choroidal vessels below atrophic areas. **a**–**d** Images of a patient with advanced AMD and geographic atrophy. Atrophy causes RPE and choriocapillaris disruption. **a** En face choriocapillaris angiogram shows displaced choroidal vessels mimicking well-formed vessels seen in macular neovascularization. **b** En face choriocapillaris structural image shows increased signal intensity in atrophic area due to RPE disruption and increased signal penetration into the choroid. **c** B-scan through atrophic area shows increased choroidal flow detection and upward displacement of choroidal vasculature below the atrophic area (white arrow). **d** B-scan without flow overlay shows choroidal hypertransmission (white arrow), a known OCT landmark for geographic atrophy

Conversely, the presence of atrophy causes RPE disruption and increased signal penetration into the choroid. En face structural images will show increased signal intensity over atrophic areas. Accompanying choriocapillaris loss leads to upward displacement of the larger choroidal vasculature, which can mimic a MNV on OCTA en face (Fig. 5a). In this situation, evaluation of the B-Scan cross section will show choroidal flow and light hypertransmission associated with atrophy (Fig. 5d) [36].

## Clinical utility of OCTA in retinal pathology

The above interpretation toolkit provides a foundation for OCTA image analysis. Understanding what OCTA features to focus on for specific disease entities can reduce interpretation times and prevent interpretation errors.

### OCTA in diabetic retinopathy

FA studies conducted by the ETDRS group and others identified vascular changes associated with DR long ago [37–39]. OCTA research reproduced these findings and furthered our understanding of the vascular changes in DR [40]. Studies comparing FA to histology reveal that FA can only visualize a fraction of the retinal vasculature [41]. This suggests that FA lacks the sensitivity to detect vascular losses seen in the early stages of DR [10]. OCTA provides high resolution images of the retinal capillary beds, and can identify early vascular changes associated with DR that FA missed. In fact, recent studies show OCTA can identify vascular changes in diabetic patients without signs of retinopathy [42, 43]. These studies suggest the potential of OCTA as a screening tool for diabetic patients.

Aside from its promise in identifying early changes in diabetic eyes, OCTA can be used as a follow-up tool for patients with DR. Currently, FA is considered the gold standard for vessel visualization in DR [44]. However, FA is time consuming and invasive, making it difficult to obtain at every patient visit. OCTA is a fast and safe way to follow vascular changes in patients with DR. Much research has been done in the field of DR and OCTA, however, there are three main findings of particular relevance to clinical practice: foveal avascular zone (FAZ) enlargement, detection of microaneurysms, and vascular changes.

#### FAZ enlargement

**Fig. 6:**
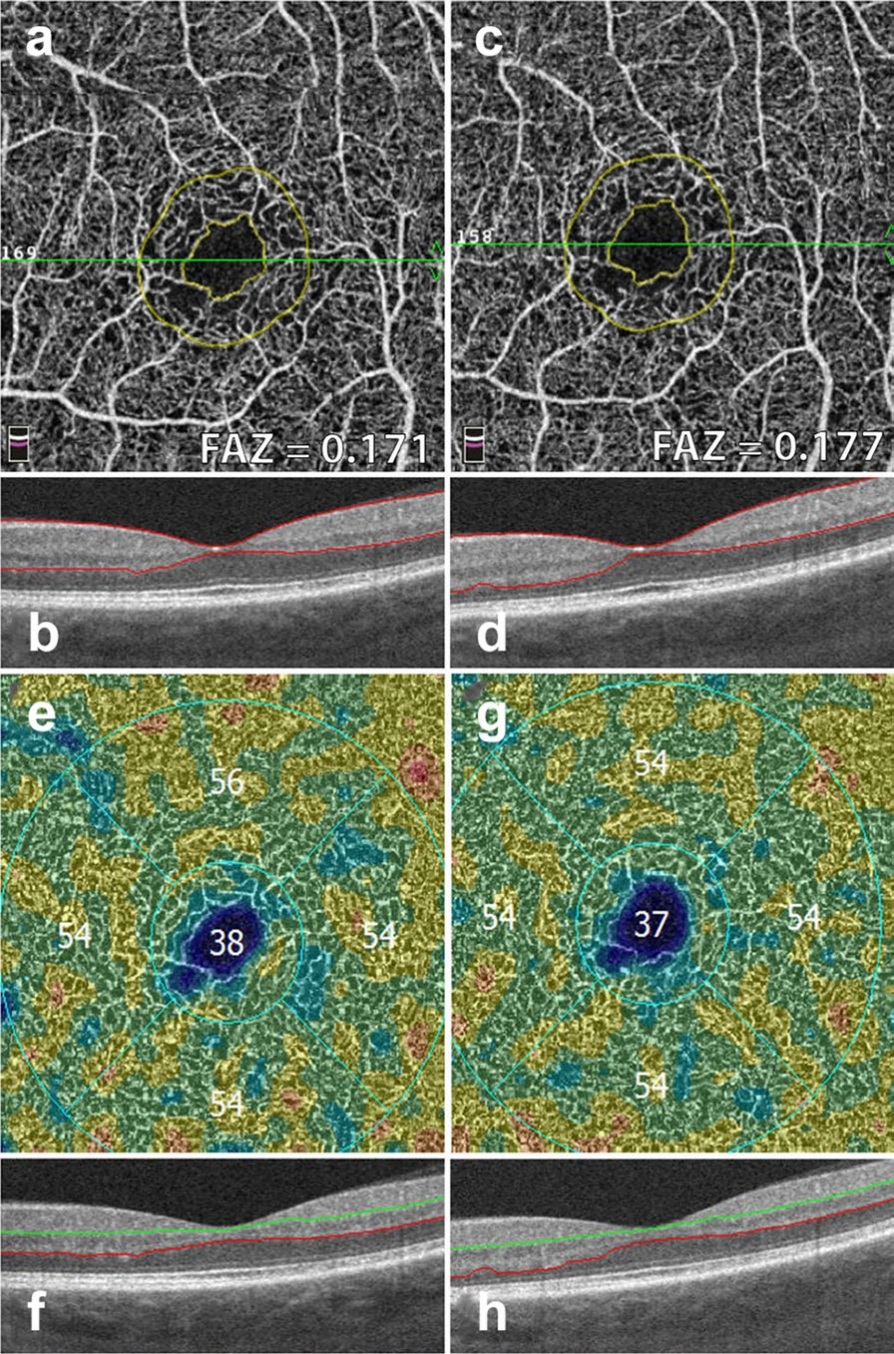
FAZ area and vessel density measurements. Example of a patient with severe non proliferative diabetic retinopathy. Images in the left column were taken 12 months prior to images in the right column. **a**, **c** Superficial retinal slab projection showing automated FAZ area measurements. Note parafoveal capillary drop out and slight increase in FAZ area over time. **b**, **d** B-scan showing corresponding segmentation boundaries. **e**, **f** Vessel density map of the deep retinal slab for the same patient and timepoints. Note slight drop in vessel density in the superior quadrant. **f**, **g** B-scan with corresponding segmentation lines

Early FA studies show that FAZ area increases with DR severity [45]. OCTA studies have replicated these findings in the full retinal layer, the superficial capillary plexus and the deep capillary plexus [46, 47]. FAZ area is considered to be a reliable and reproducible metric between readers in both normal eyes and eyes with DR [48, 49]. Though FAZ can be assessed at both the deep capillary plexus and superficial capillary plexus, there is evidence to suggest that the retinal plexuses meet at the edge of the FAZ and it is, therefore, preferable to measure the FAZ on the superficial or the full retinal depth projection [50]. Assessing FAZ area on the full retinal depth projection obviates the need for segmentation and accompanying errors, increasing the measurement’s reproducibility [51, 52]. Clinically, assessing FAZ enlargement over time can be a useful tool for following DR progression (Fig. 6).

#### Microaneurysms

**Fig. 7:**
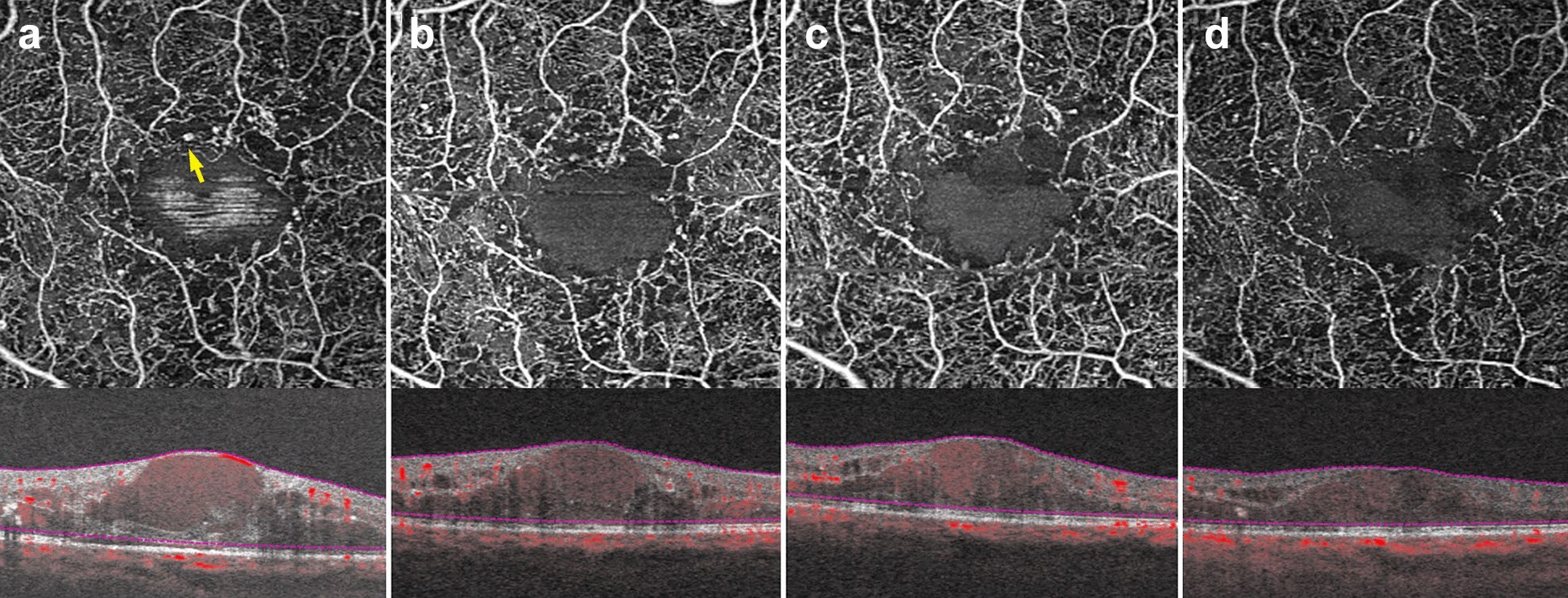
Microaneurysm response to anti-VEGF therapy. **a**–**d** 3 x 3 mm images of the full retinal depth projection of a patient treated with anti-VEGF therapy for diabetic macular edema. Images were taken 3 months apart over a 10-month period. **a** Yellow arrow points to an example of a microaneurysm. Note reduction in the number of parafoveal microaneurysms over time with concurrent improvement of edema

Microaneurysms (MA) are a landmark finding in DR and are associated with an increased risk of progression to advanced disease stages [39, 53]. Studies show that OCTA can visualize approximately 60% of the MA identified on FA [46, 54]. MA are focal dilations of retinal capillaries. Flow can slow within these capillary dilations and drop below the OCTA detection threshold [1]. Prior to the advent of anti-VEGF agents, identifying the location of all MA was essential to proper laser treatment. As anti-VEGFs are increasingly used to treat complications of DR, including DME and PDR, OCTA can be used to track the response of MA to treatment (Fig. 7) [55, 56]. Though MA tracking has little utility in assessing treatment response in DME, the presence of increased MAs and ischemia may mark areas at higher risk of DME recurrence [57].

#### Vascular changes

Research has uncovered associations between vascular changes seen on OCTA and increasing DR severity [58]. Studies point to significant vessel loss in patients with DR as compared to healthy controls [59]. A negative association between vessel density and increasing DR severity at both the superficial and deep capillary plexuses has been repeatedly identified [60–62]. Though quantitative vessel metrics on OCTA have been shown to have low reproducibility between devices, certain OCTA platforms provide color coded perfusion maps based on the vessel density of normal eyes [52]. These maps can help providers track DR severity and assess vessel loss in conjunction to disease progression (Fig. 6) [3].

**Fig. 8:**
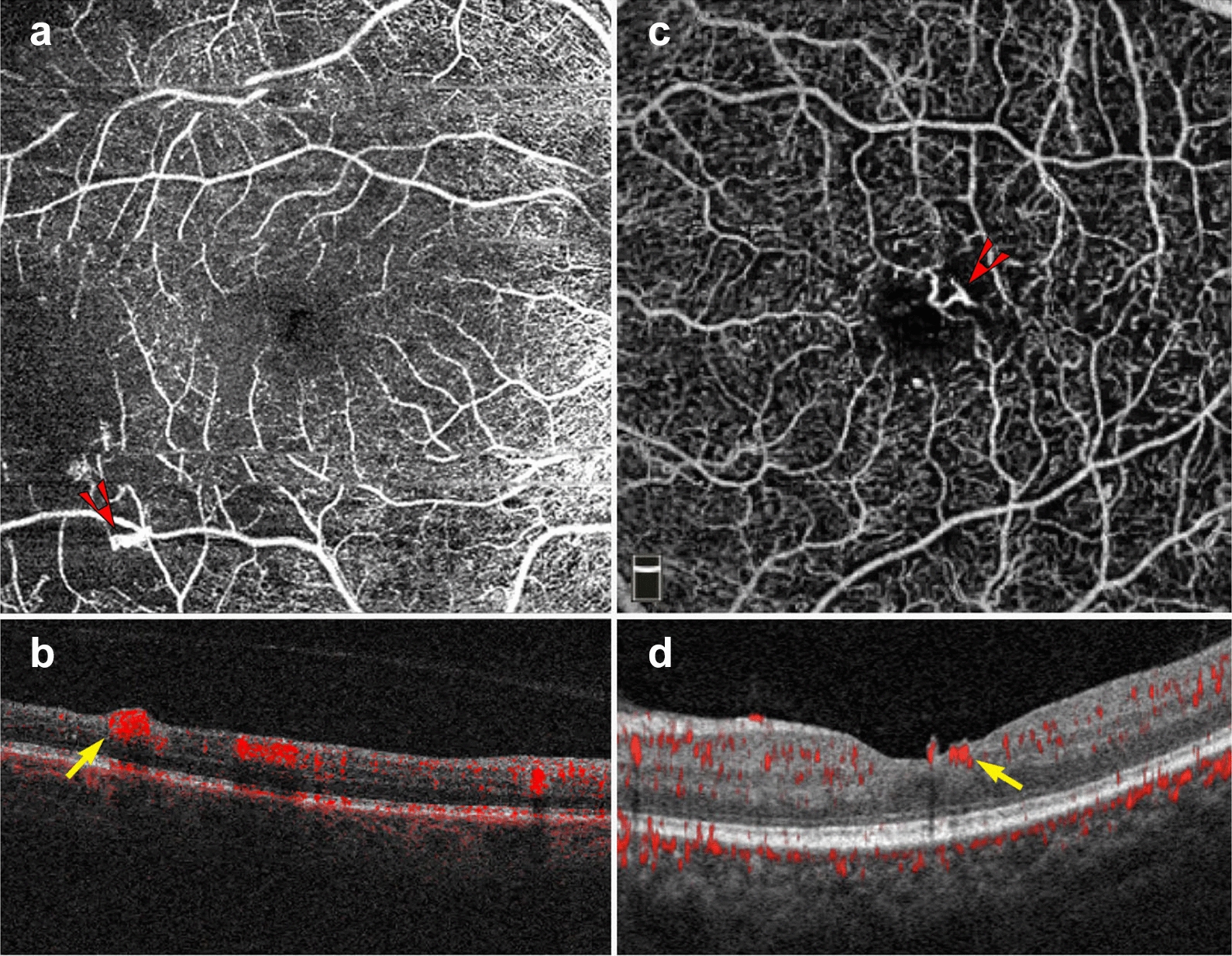
Proliferative diabetic retinopathy. **a**–**d** 6 x 6 mm images of the full retinal depth projection of two patients with proliferative diabetic retinopathy (PDR). **a**, **c** En face view of PDR, red arrowheads point to areas of neovascularization. **b**, **d** Corresponding B-scans show flow at the vitreoretinal interface (yellow arrows)

In addition to the vascular changes associated with non-proliferative DR, OCTA is also useful in the clinical management of PDR. PDR is an advanced stage of DR that can lead to vision threatening complications such as hemorrhage and tractional retinal detachment [63]. Neovascular changes associated with PDR can been visualized on OCTA (Fig. 8) [2]. OCTA can identify neovascular growth and follow its regression in response to anti-VEGF treatment [64].

#### Widefield OCTA imaging

Though original OCTA devices imaged only the central macula, newer OCTA technology offers a wider field of view. New swept source devices have larger scan areas of 15 × 9 mm and 12 × 12 mm and can visualize a greater portion of the posterior retina when compared to original devices offering only 3 × 3 and 6 x 6 mm scans [65]. As the field of view expands, however, resolution decreases and acquisition times lengthen. To offset resolution loss, montage protocols that offer fields of view of up to 100 degrees without sacrificing resolution have been developed. Though the 100-degree field of view offered by wide-angle OCTA is smaller than the 200-degree field of view offered by ultra-wide field (UWF) FA, OCTA images display higher levels of vascular detail and offer three-dimensional data not available on FA [66].

An increased field of view allows for improved imaging of the posterior pole, an important characteristic when assessing peripheral DR pathology. Wide-field SS-OCTA can be utilized to detect PDR and track response to panretinal photocoagulation. When compared to UWF FA, wide-field SS-OCTA was equivalent for the identification of PDR progression and regression, but SS-OCTA offered more detailed visualization of vascular changes [67]. Studies comparing UWF FA and wide-angle OCTA have found both imaging modalities equivalent for the detection of capillary drop out and retinal neovascularization in DR [68, 69]. These findings are encouraging and suggest wide-angle OCTA may provide a non-invasive alternative for vascular monitoring in DR.

### OCTA in AMD and MNV

OCTA has proven invaluable for the study of AMD. The ability of OCTA to visualize the choriocapillaris in intermediate AMD and later stages of the disease has shed light onto the etiology of this vision threatening disease. Though OCTA has provided great investigative insight into the changes underlying dry AMD, the clinical use of OCTA in AMD is largely limited to the diagnosis of neovascular or wet AMD. Based on structural OCT, there are three types of MNV associated with wet AMD. Type 1 MNV lies below the RPE, type 2 MNV lies between the RPE and the retina, and type 3 MNV arises from the superficial retinal vasculature and grows downwards toward the neurosensory retina and eventually the choriocapillaris [70].

**Fig. 9:**
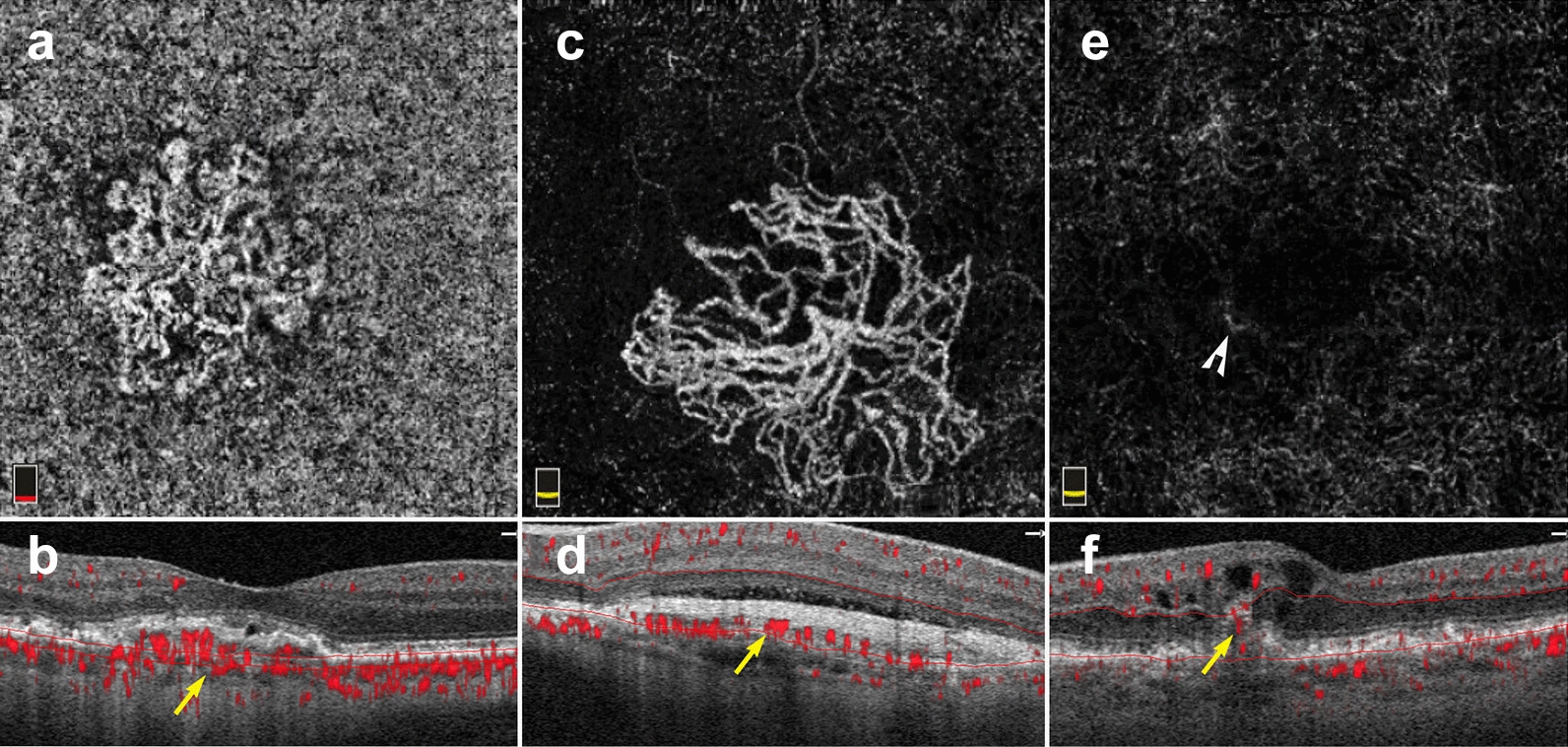
Macular neovascularization types. **a**–**f** 3 x 3 mm en face images and corresponding B-scans for the 3 MNV types seen in wet AMD. Segmentation was manually adjusted to reveal the best en face view for each lesion. **a**,**b** Type 1 MNV: distinct vascular inlets at the level of the choriocapillaris are seen on en face (**a**), B-scan reveals flow below the RPE (**b**, yellow arrow). **c**, **d** Type 2 MNV: en face view shows vascular branching suspicious for an MNV (**c**), B-scan shows flow between the retina and the RPE (**d**, yellow arrow). **e**, **f** Type 3 MNV: en face view shows a small bright tuft (**e**, white arrowhead), B-scan view shows intraretinal flow trailing downwards, towards the choriocapillaris (**f**, yellow arrow). Of note, type 3 MNV are very difficult to visualize on en face and often appear only as small, white tufts as depicted in the above example (**e**)

The ability to register flow information with structure makes OCTA a useful tool for MNV diagnosis and classification. En face OCTA images can depict the vascular networks associated with each type of MNV, while B-scans reveal their exact location within the retina (Fig. 9). OCTA can detect non-exudative MNV that were previously unnoticed. These lesions are generally type 1 MNV that lie below the RPE and are associated with a ‘double layer sign’, flat irregular, low lying PEDs that may go unnoticed in a superficial evaluation of OCT scans [71]. Studies suggest high sensitivities and specificities for the identification of type 1 and type 2 MNV on OCTA [72, 73]. However, MNV detection sensitivity drops precipitously with signal reduction secondary to hemorrhage and large PEDs, so OCTA should be used with caution in this context [74].

Visualization of MNV on OCTA is helpful for diagnosis and classification. However, the clinical utility of OCTA for tracking MNV response to anti-VEGF treatment remains unclear. Several studies reveal MNV shrinking on OCTA after anti-VEGF treatment, albeit often without complete resolution [75–78]. A study by Lumbroso and colleagues showed that MNV reach maximal size reduction 1 week after intravitreal anti-VEGF injection, and progressively increase in size thereafter. Interestingly, this work also showed that MNV expansion predates MNV re-exudation [79]. Tracking MNV changes on OCTA to assess treatment response has not been proven to improve clinical outcomes. Thus, decisions regarding re-treatment are currently driven by visual acuity, clinical examination, presence of exudation and structural OCT changes.

Interpretation pitfalls discussed earlier in this review must be carefully considered when studying AMD and MNV on OCTA. Important points to remember include:

1. AMD features, such as drusen, geographic atrophy and MNV, can affect automatic device segmentation. Assessment of segmentation lines on B-scan is necessary prior to interpretation.
2. MNV lesions should always be interpreted in the context of their corresponding B scan. MNV types lie at different retinal depths. The preset slabs available on commercial OCTA devices often do not provide an ideal en face view of MNV lesions. Instead, manual slab selection can be used to fully encompass the lesion and improve en face visualization of MNV vessel networks.
3. Reflective surfaces such as the RPE above type 1 MNV and drusen can lead to projection of flow from overlying retinal vasculature. B-scan assessment can help highlight this type of artifact and reduce false-positive findings.

### OCTA and other pathologies

The use of OCTA in clinical practice is not limited to DR and AMD. OCTA is useful in a range of retinal pathology including retinal vascular disease, retinal inflammatory disease, retinal tumors and retinal hereditary dystrophies.

In venous occlusive diseases, such as central and branch retinal vein occlusions, OCTA can identify areas of non-perfusion as well as collateral vessel formation [80, 81]. Manual slab selection provides information regarding the affected retinal vascular layers and the location of new collateral vessels. In retinal inflammatory disease, OCTA can detect vascular changes associated with inflammation including retinal hypo-perfusion in occlusive vasculitis and increased vessel density around the optic nerve head in uveitis [8, 82]. Moreover, OCTA aids in the identification of choroidal neovascularization secondary to inflammation (iCNV). Since inflammatory CNVs can mimic other types of inflammatory non-neovascular lesions, OCTA can detect flow within iCNVs and lead to prompt diagnosis and treatment [83].

OCTA has led to novel vascular findings in retinal hereditary dystrophies. For example, patients with choroidemia have reduced macular choriocapillaris density on OCTA when compared to controls, a finding not previously recognized by standard angiography [84]. OCTA was found to be superior to FA for the detection of CNV in patients with Best Vitelliform Macular Dystrophy, due to improved signal penetration through vitelliform material [85]. The use of OCTA for the vascular assessment of ocular tumors has also been extensively studied [86]. Vascular findings on OCTA, such as avascular areas and vascular abnormalities, might be of clinical utility in the differentiation of choroidal nevi from small choroidal melanomas [87]. In retinal capillary hemangioblastomas, OCTA can be used for diagnosis and assessment of treatment response to anti-VEGF therapy [88].

## The future of OCTA

OCTA is evolving from a research tool to a clinically useful imaging modality. Its ease of use, depth resolution, and ability to analyze microvasculature provide a wealth of information beyond what other retinal vascular imaging offers.

The advent of swept source OCTA technology allowed for clear visualization of choroidal vasculature [15]. As laser technology continues to improve, newer devices with increased signal penetration will visualize choroidal vascular detail at greater depths. Further, faster scanning speeds will allow for wider and wider fields of view. This could have lasting implications for the assessment and risk stratification of a host of diseases from choroidal tumors and choroidal neovascularization, to retinal vascular disease.

Along with these hardware improvements, technical innovations in OCTA signal processing have reduced the presence of artifacts in prototype devices [28]. Device implementation of image alignment and tracking for longitudinal data will better serial assessment of progressive retinal diseases, including AMD and DR. As these improvements become commercially available, the clinical applicability and utility of OCTA will continue to expand and could have lasting implications in the way we image the retinal vasculature. Moreover, application of machine learning to OCTA will yield insights into new pathologic signals that were previously overlooked that could greatly impact the clinical management of retinal disease [89, 90].

The future of OCTA is promising, but clinicians require appropriate training to derive meaning out OCTA data and to avoid interpretation errors. This review provides eye care professionals with a toolkit for OCTA interpretation in the clinical setting. A solid understanding of the artifacts associated with OCTA imaging along with a step-wise approach to image interpretation offers the flexibility to apply OCTA analysis to a wide range of ocular pathology.

## Data Availability

Not applicable.

## Abbreviations

OCTA: Optical coherence tomography angiography
FA: Fluorescein angiography
ICGA: Indocyanine green angiography
AMD: Age-related macular degeneration
DR: Diabetic retinopathy
OCT: Optical coherence tomography
SD-OCT: Spectral domain optical coherence tomography
SD-OCTA: Spectral domain optical coherence tomography angiography
SS-OCTA: Swept source optical coherence tomography angiography
Anti-VEGF: Vascular endothelial growth factor antagonists
RPE: Retinal pigment epithelium
DME: Diabetic macular edema
MNV: Macular neovascularization
ORCC: Outer retinal and choriocapillaris
PED: Pigment epithelial detachment
FAZ: Foveal avascular zone
MA: Microaneurysm
PDR: Proliferative diabetic retinopathy
CNV: Choroidal neovascularization
